# Transcriptome changes in *Fusarium verticillioides* caused by mutation in the transporter-like gene *FST1*

**DOI:** 10.1186/s12866-015-0427-3

**Published:** 2015-04-25

**Authors:** Chenxing Niu, Gary A Payne, Charles P Woloshuk

**Affiliations:** Department of Botany and Plant Pathology, Purdue University, 915 W. State Street, West Lafayette, IN 47907-2054 USA; Department of Plant Pathology, North Carolina State University, 851 Main Campus Drive, Raleigh, NC 27695-7567 USA

**Keywords:** *Fusarium verticillioides*, RNA-seq, Transcriptome, *FST1*, Fumonisin, Mycotoxins, Hydrophobin, Virulence, Reactive oxygen species, Transporter, Sensor

## Abstract

**Background:**

*Fusarium verticillioides* causes an important seed disease on maize and produces the fumonisin group of mycotoxins, which are toxic to humans and livestock. A previous study discovered that a gene (*FST1*) in the pathogen affects fumonisin production and virulence. Although the predicted amino acid sequence of FST1 is similar to hexose transporters, previous experimental evidence failed to prove function.

**Results:**

Three new phenotypes were identified that are associated with the *FST1* mutant of *F. verticillioides* (Δfst1), namely reduction in macroconidia production, increased sensitivity to hydrogen peroxide, and reduced mycelial hydrophobicity. A transcriptome comparison of the wild type and strain Δfst1 grown on autoclaved maize kernels for six days identified 2677 genes that were differentially expressed. Through gene ontology analysis, 961 genes were assigned to one of 12 molecular function categories. Sets of down-regulated genes in strain Δfst1 were identified that could account for each of the mutant phenotypes.

**Conclusion:**

The study provides evidence that disruption of *FST1* causes several metabolic and developmental defects in *F. verticillioides. FST1* appears to connect the expression of several gene networks, including those involved in secondary metabolism, cell wall structure, conidiogenesis, virulence, and resistance to reactive oxygen species. The results support our hypothesis that *FST1* functions within the framework of environmental sensing.

## Background

*Fusarium verticillioides* (telemorph, *Gibberella moniliformis*), which is present in most maize fields, can be an asymptomatic endophyte or the causal agent of seedling, stalk, ear, and kernel diseases [1]. The pathogen produces fumonisins, a group of structurally related polyketide mycotoxins, during colonization of maize kernels. Ingestion of fumonisin B1 (FB1), the most predominant fumonisin analog, can result in leukoencephalomalacia in horses and pulmonary edema in swine. The mycotoxin also has been implicated in human diseases, including cancer and birth defects [2]. Guidelines for maximum fumonisin levels in human food and animal feeds have been established worldwide [1]. Furthermore, economic losses associated with fumonisin contamination in maize exports by the three major maize-exporting nations (US, China and Argentina) were estimated at $100 million annually [3] with the US losses alone at nearly $40 million annually [4].

Recent publications describe the complexity of genes that influence regulation of fumonisin biosynthesis. Pathway-specific activator *FUM21* (FVEG_14633), which controls transcription of the cluster of *FUM* genes [5], was shown to increase when *F. verticillioides* was treated with the histone deacetylase inhibitor chostatin A [6]. These results support evidence that histone modification plays an important role in the epigenetic regulation of fumonisin production [7,8]. There are several intriguing reports indicating that environmental conditions (nutrients and pH) also affect the transcription of *FUM* genes and FB1 production. Expression of the nitrogen utilization gene *AREA* (FVEG_02033) was found to be responsible for repression of FB1 production by ammonium [9]. Under repression conditions, AREA is hypothesized to bind to GATA sequences in the promoters of the *FUM* genes. Generally, acidic conditions favor FB1 production [10]. Experimental evidence indicates that *PACC* (FVEG_05393), which has homology to the alkaline-activator gene *PACC* in *A. nidulans* [11], inhibits FB1 production and *FUM1* (FVEG_00316) transcription at pH 8 [12]. Finally, carbon source and availability, especially amylopectin, greatly affect FB1 biosynthesis [13]. Studies on carbon utilization have led to the identification of two genes, *HXK1* (FVEG_00957) and *FST1* (FVEG_08441). HXK1, a putative hexose kinase was shown to be required for fructose metabolism [14]. Strains without a functional HXK1 also produced less FB1 and were less virulent on maize kernel than the wild type (WT). The function of *FST1* is the focus of the current study.

*FST1* was identified through a comparative analysis of genes expressed in colonized maize germ and endosperm tissues [15]. Of 50 putative sugar transporter genes represented on a microarray, *FST1* was one of six genes identified as highly expressed during fungal growth in endosperm tissue compared to germ tissue [15]. Expression of *FST1* was also reduced in a *F. verticillioides* strain with a disrupted *ZFR1* gene, a putative Zn_2_Cys_6_ transcription factor [15]. *FST1* encodes a 574-amino-acid protein with 12 putative transmembrane domains. Heterologous expression of *FST1* in yeast system failed to show hexose transporter activity [16]. Disruption of *FST1* in *F. verticillioides* resulted in reduced virulence and FB1 production [15,16]. The reduced virulence phenotype in inoculated kernels was manifested as slower growth and rot symptoms when compared to the WT [16]. When inoculated onto autoclaved kernels or synthetic media, mutant growth was the same as WT [15]. In contrast, the mutant failed to produce FB1 on either living or dead kernels.

In the current study, we describe three new phenotypes attributed to a non-functional *FST1*. Furthermore, we describe the effects of *FST1* on whole genome expression by comparing the transcriptomes of the WT and ∆fst1 strains of *F. verticillioides* grown on autoclaved maize kernels. The results support our hypothesis that *FST1* has a regulatory function that globally impacts gene expression.

## Results

### Macroconidia production and sensitivity to H_2_O_2_

Wild type *F. verticillioides* produces primarily microconidia and very few macroconidia. When grown on carnation leaf agar (CLA) medium, higher numbers of macroconidia are produced on the leaves. We found that strain ∆fst1 produced only 10% as many macroconidia as the WT (Table 1). In the complemented strain fst1-comp, macroconidia production approached WT levels (83%). There were no measurable differences between ∆fst1 and WT in the production or morphology of microconidia, conidiophores, or microconidal chains.

**Table 1:** **Effect of Δfst1 on conidiation**

**Strain**	**Macroconidia** ^**a**^	**Microconidia**
WT	90 ± 7	1,252 ± 118
Δfst1	9 ± 3*	1,052 ± 123
Fst1-comp	75 ± 7	1,603 ± 163

^a^Macroconidia and microconidia values are mean number of conidia per carnation leaf from nine replicates at 7dpi +/− standard error.

^*^Indicates significant difference from other values in column. (α = 0.05).

**Figure 1:**
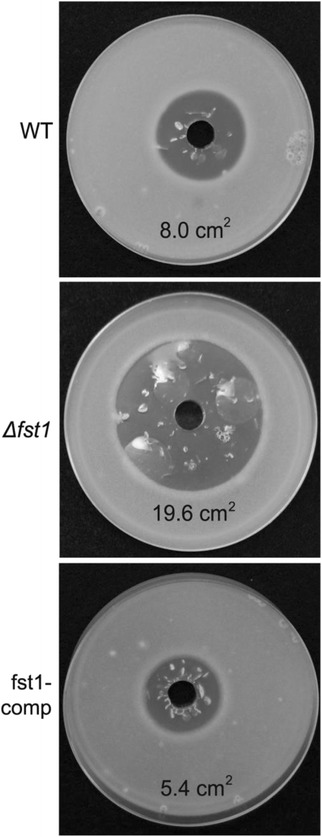
Resistance to hydrogen peroxide assay. Conidia of wild type, ∆fst1, and fst1-comp were suspended into molten PDA medium. After 24 hours, 15% hydrogen peroxide solution was added to a well cut into each culture. Photograph was taken after two days of incubation. Each plate is labeled with the mean area of inhibition (clear zone) for three replicates. The standard errors were 0.01 cm^2^ or less.

To determine if the reduced growth phenotype of ∆fst1 mutants grown on living kernels was associated with increased sensitivity to reactive oxygen species, we evaluated growth of ∆fst1 mutants on agar plates amended with hydrogen peroxide. Δfst1 was found to be more sensitive than WT, and the differences were most pronounced at 15% H_2_O_2_ (v/v) (Figure 1). The zone of the inhibition for strain Δfst1 was 2.4 and 3.6 times larger than that of the WT and strain fst1-comp, respectively.

### Analysis of transcriptome

RNA isolated from four biological replicates of Δfst1 and WT were sequenced, which resulted in a total of over 836 million reads (Table 2). Approximately 752 million (90%) of the total reads uniquely mapped to the reference genome of *F. verticillioides*. Results from the mapping indicated that of the 15,869 annotated genes of *F. verticillioides*, 14,769 and 14,893 genes were expressed (RPKM > 0) in Δfst1 and WT, respectively. To identify differentially expressed genes, a pairwise t-test was made between the expression data of WT and strain Δfst1. The expression of 2,677 genes was found to be significantly different (P value < 0.01) with an absolute fold difference greater than two. Of these, 1,081 (40.4%) genes were up-regulated in Δfst1 and 1,596 (59.6%) genes were down-regulated. Also, we identified 373 and 249 genes that were uniquely expressed in WT and Δfst1, respectively. Expression of four putative tubulin and three putative elongation factor genes was similar in both strains and not statistically different (Table 3), indicating that the mutation in strain Δfst1 did not impact expression of these house-keeping genes. The differentially expressed genes were functionally categorized based on gene ontology (GO) annotation and placed into one of 13 groups (Table 4). Two-thirds of the genes were classified as encoding hypothetical proteins.

**Table 2:** **Summary of RNAseq data from Illumina sequencing** ^**a**^

**Sample Name**	**Total Reads**	**Percent Mapped**
Fst1-1	89,159,452	91
Fst1-2	90,508,852	91
Fst1-3	120,993,332	90
Fst1-4	122,219,758	89
WT1	111,712,848	89
WT2	96,499,202	90
WT3	106,782,286	90
WT4	98,144,940	90

^a^Paired-end data were trimmed to remove low quality sequence, and reads less than 30 nt were filtered out of the final data sets. These data were mapped to the *F. verticillioides* reference genome with CLC Genomic Workbench 7.0.4 software.

**Table 3:** **Expression of tubulin and elongation factor (EF) genes during colonization of autoclaved maize kernels by strains Δfst1 and WT** ^**a**^

**FVEG ID**	**Gene name**	**WT**	**Δfst1**
00855	Tubulin alpha chain	198^b^	172
00557	Tubulin alpha chain	195	220
05512	Tubulin beta chain	162	183
02785	Tubulin gamma chain	23	21
02381	EF 1-alpha	2345	2360
04016	EF 1-alpha	25	23
09131	EF 2	9	8

^a^Data were collected from cultures grown for 6 days on autoclaved maize kernels.

^b^Data are the mean RPKM values of four biological replicates.

**Table 4:** **Molecular function ontology of differentially expressed genes in WT and Δfst1 during colonization of autoclaved maize kernels** ^**a**^

**Molecular Function** ^**b**^	**Up in ∆fst1**	**Down in ∆fst1**	**Not expressed in WT**	**Not expressed in** ***∆fst1***
Hydrophobins	1	3	0	0
Fumonisin biosynthesis	0	12	0	0
Decarboxylases	6	9	2	0
Reductases	20	18	1	3
Kinases	18	32	3	3
Peptidases and Proteases	13	39	1	5
Integral Membrane Proteins	40	22	3	7
Hydrolases	42	64	5	12
Transcription Factors	49	66	5	3
Dehydrogenases	48	78	6	7
Oxidases	66	123	7	21
Transporters	97	94	16	19
Hypothetical Proteins	681	1035	200	293
Total	1081	1596	249	373

^a^Data were collected from cultures grown for 6 days on autoclaved maize kernels.

^b^Ontology assignments based on top BLAST from Blast2GO analysis.

### FUM gene cluster

One of the functional categories included the genes involved in fumonisin biosynthesis (Table 4). Expression of all 15 *FUM* genes was measurable in both the WT and strain Δfst1 (Table 5). Statistical testing indicated that 12 genes had significantly different (P value < 0.01, absolute fold change > 2) expression between the two strains. *FUM* 11, 16 and 21 with P values of less than 0.02 did not meet the criteria for statistical significance. All *FUM* genes were down-regulated in strain Δfst1, with at least 4-fold reduction in expression compared to WT. The greatest difference was in the expression of *FUM1* (polyketide synthase gene), which was reduced more than 37-fold in the mutant. Analysis of expression by qPCR verified that both *FUM*1 and *FUM*21 expression was less in Δfst1 compared to WT (Table 6).

**Table 5:** **Comparison of expression of FUM genes in wild type (WT) and Δfst1** ^**a**^

**Gene name**	**FVEG ID** ^**b**^	**P value** ^**c**^	**Log**_**2**_ **fold change** ^**d**^
*FUM1*	00316	0.004*	−5.21
*FUM2*	00323	0.008*	−3.15
*FUM3*	00320	0.003*	−3.41
*FUM6*	00317	0.003*	−3.63
*FUM7*	00319	0.002*	−2.96
*FUM8*	14634	0.005*	−3.54
*FUM10*	00321	0.004*	−3.60
*FUM11*	00322	0.012	−3.06
*FUM13*	00324	0.002*	−3.59
*FUM14*	00325	0.006*	−3.20
*FUM16*	00326	0.019	−2.74
*FUM17*	00327	0.007*	−3.03
*FUM18*	00328	0.008*	−2.76
*FUM19*	00329	0.003*	−2.90
*FUM21*	14633	0.012	−2.20

^a^Data were collected from cultures grown for 6 days on autoclaved maize kernels.

^b^Fusarium Comparative Database (Broadinstitute.org).

^c^P value from pairwise t-test of mean RPKM of Δfst1 and WT. Values with* meet the criteria of P < 0.01.

^d^Values derived from the mean RPKM of Δfst1/WT.

**Table 6:** **Expression of selected genes in strain Δfst1 relative to expression in wild type (WT) of** ***F. verticillioides***

**Gene Name**	**FVEG ID** ^**a**^	**Relative Expression** ^**b**^	
		**Autoclaved Kernels**	**Living Kernels**
*FUM1*	00316	−33.3 (−25.8, − 48.5)	
*FUM21*	14633	−9.4 (−7.6, − 11.7)	
*POD1*	10866	−3.9 (−3.7, − 4.1)	−2.0 (−2.0, − 2.0)
*POD3*	12884	−86.7 (−73.3, − 102.6)	−102.1 (−71.0, −147.0)
*POD4*	12465	−119.1 (−115.8, − 122.5)	−4.1 (−4.1, − 4.2)
*CAT1*	05529	−12.0 (−10.1, − 14.2)	−32.4 (−21.8, − 47.1)
*CAT2*	12611	3.7 (3.3, 4.0)	2.2 (1.9, 2.4)
*CAT3*	11955	1.5 (1.5, 1.6)	4.9 (4.8, 4.9)
*HYD3*	06538	−76.0 (−73.3, − 78.7)	
*HYD7*	09843	33.7 (31.8, 35.7)	
*ITR1*	06504	−34.4 (−23.5, − 50.4)	
*FLF1*	12826	−2.9 (−2.8, − 3.0)	
*TFS1*	06118	−2.6 (−2.4, − 2.8)	
*AGD1*	14136	−22.1 (−20.5, − 24.0)	

^a^Fusarium Comparative Database (Broadinstitute.org).

^b^Expression was measured by quantitative reverse-transcriptase polymerase chain reaction (qPCR). RNA from biological replicate samples of WT or Δfst1 was pooled for cDNA synthesis, and three technical replicates were analyzed for each gene. Expression of *TUB1* (FVEG_04081) was used to normalize data. For each gene, values represent fold differences in Δfst1 with WT expression set at a value of 1. Expression of each gene was calculated as 2^ΔΔCt^. Range of expression is in parentheses equals 2 ^ΔΔCt-s^, 2 ^ΔΔCt+s^, where s equals the standard deviation of the 2^ΔΔCt^ value.

### Hydrophobin genes

**Figure 2:**
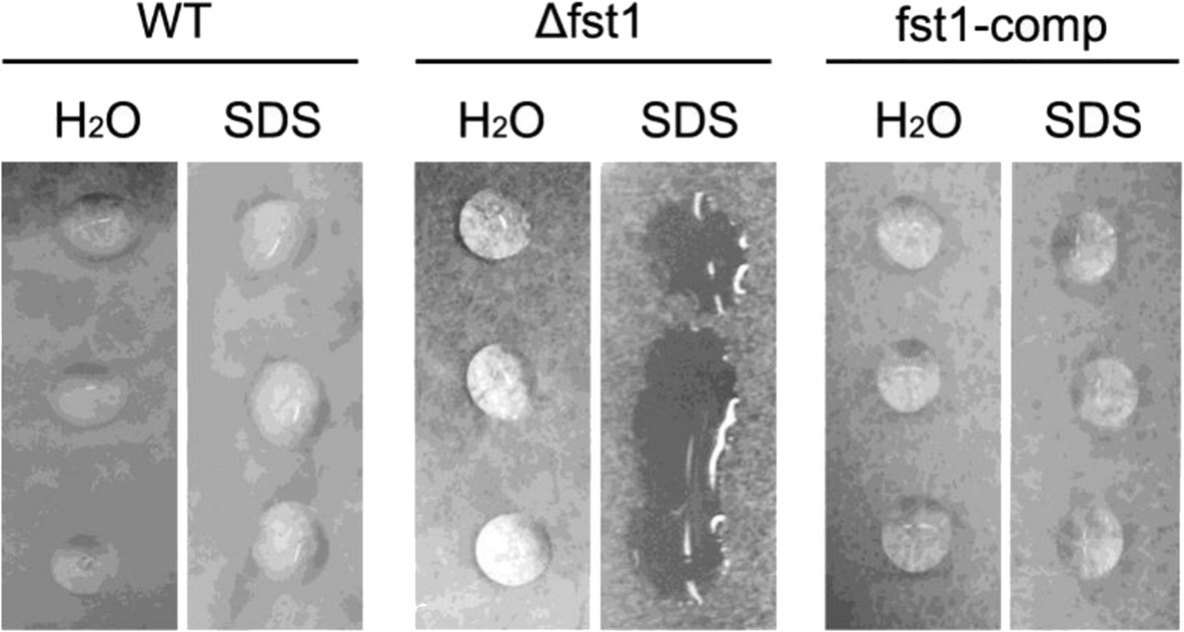
Mycelial hydrophobicity assay. Cultures of wild type and ∆fst1 were grown for six days on PDA medium. Photograph was taken 30 min after placement of droplets (10 μl) of water and SDS solution on the colony surface.

Eight hydrophobin genes have been identified in *F. verticillioides, HYD1-8* [17,18]. Hydrophobins are a group of small, cysteine-rich proteins expressed in filamentous fungi, which form a hydrophobic/hydrophilic interface on the surface of hyphae and conidia. RNAseq analysis revealed significant differences in the expression of *HYD3*, *HYD*4, *HYD*5 and *HYD*7, with a 49.5-fold, 4.4-fold, 6.3-fold reduction and 54-fold increase, respectively, in strain Δfst1 (Table 7). The differences in expression of *HYD3* and *HYD7* were verified by qPCR (Table 6). The expression of *HYD1*, *HYD2, HYD6* and *HYG8* was not significantly different. To test for defects in hydrophobicity, droplets of water or a detergent solution were placed on fungal mycelium of WT, Δfst1, and the complemented strain fst1-comp. For all three strains, droplets of water maintained a spherical shape for more than 30 min. Droplets of detergent solution on the WT and strain fst1-comp also remained intact (Figure 2). However, on strain Δfst1, the droplet spread out over the surface of the mycelium, indicating a defect in hydrophobicity.

**Table 7:** **Comparison of hydrophobin (** ***HYD*** **) genes during colonization of autoclaved maize kernels by strains Δfst1 and WT** ^**a**^

**Gene name**	**FVEG ID** ^**b**^	**P value** ^**c**^	**Log**_**2**_ **fold change** ^**d**^
*HYD1*	03689	-^e^	-
*HYD2*	03685	-	-
*HYD3*	06538	0.0001	−5.63
*HYD4*	01575	0.0019	−2.13
*HYD5*	07695	0.0083	−2.65
*HYD6*	01573	-	-
*HYD7*	09843	0.0036	5.76
*HYD8*	10008	-	-

^a^Data were collected from cultures grown for 6 days.

^b^Fusarium Comparative Database (Broadinstitute.org).

^c^P value from pairwise t-test of mean RPKM of Δfst1 and WT.

^d^Values derived from the mean RPKM of Δfst1/WT.

^e^Data not significant (P value > 0.01).

### Transcription factors

Ma et al. [19] predicted 683 putative transcription factor (TF) genes in *F. verticillioides* and Wiemann et al. [20] predicted 640. Of these predicted TF, our analysis identified 115 differentially expressed (Table 4). Transcription factors in fungi have been classified into 61 families [21], and we found that 108 of the differentially expressed TF genes were in 12 of the 61 families. Most (80%) of the TFs were C_2_H_2_ zinc finger (16 genes) and Zn_2_Cys_6_ (76 genes). *FUM21* is classified in the Zn_2_Cys_6_ family and its expression in Δfst1 was 4.6-fold less compared to that of the WT (Table 5). However, its P-value (0.012) was just outside the threshold we selected for statistical testing.

### Transporters

A total of 191 differentially expressed genes and 35 genes in the uniquely expressed category were classified as transporters (Table 4). We separated the 191 differentially expressed transporter genes into seven categories: ABC transporter, amino acid related transporter, ammonium related transporter, mineral/ion related transporter, major facilitator superfamily, sugar transporter, and uncategorized (Table 8). In the categories for sugar and ammonium-related transporters, considerably more genes were up-regulated in strain Δfst1. In contrast, most of the differentially expressed genes in the ABC and mineral/ion transporter categories were down-regulated. Expression of one putative inositol transporter (*ITR1* FVEG_06504) was decreased by 19-fold in strain Δfst1 compared to WT, which was verified by qPCR analysis (34-fold) (Table 6).

**Table 8:** **Classification of putative transporter genes differentially expressed during colonization of autoclaved maize kernels by wild type (WT) and strain Δfst1** ^**a**^

**Transporter Type** ^**b**^	**Up in Δfst1**	**Down in Δfst1**
Amino acid related	14	10
ABC	1	9
Ammonium related	4	0
Mineral/ion related	9	19
Sugar	30	7
Major facilitator superfamily	23	28
Uncategorized	16	21

^a^Data were collected from cultures grown for 6 days on autoclaved maize kernels.

^b^Ontology assignments based on top BLAST from Blast2GO analysis.

### Oxidases

A total of 189 of the differentially expressed genes were categorized with putative oxidase functions (Table 4). Compared to the WT, two-thirds of these genes exhibited reduced expression in strain Δfst1 and the other third were expressed at higher levels. Additionally, the expression of 21 oxidase genes was only measured in the WT and seven only in Δfst1. A word-search of the *F. verticillioides* genome database identified 30 putative peroxidase and seven catalase genes, and ten of these genes were differentially expressed. The peroxidase genes (*POD1* FVEG_10866; *POD3* FVEG_12884; *POD4* FVEG_12465; FVEG_04790) were all down-regulated as much as 100-fold in strain Δfst1 compared to WT. Four catalase genes (*CAT1* FVEG_05529; FVEG_05976; FVEG_03348; FVEG_05591) also were down-regulated in Δfst1. Expression of the putative catalases *CAT2* (FVEG_12611) and *CAT3* (FVEG*_*11955) was up-regulated 4-fold and 2-fold, respectively, in strain Δfst1. We used qPCR analysis to measure the expression of peroxidases and catalases in both autoclaved kernels and infected living kernels. In autoclaved kernels, expression of three peroxidase genes (*POD1, POD3* and *POD4*) and three catalase genes (*CAT1, CAT2* and *CAT3*) were found to be similar to expression indicated by the RNAseq results (Table 6). qPCR analysis of the inoculated living kernels indicated similar effects on expression of the peroxidases and catalases (Table 6).

### Secretome

The Fungal Secretome Database (http://fsd.riceblast.snu.ac.kr) lists 1412 genes in *F. verticillioides* that encode putative secreted proteins, and a comparison with the updated reference genome at the Broad Institute matched 1402 of these genes. Our RNAseq analysis indicated that 1310 and 1330 of the genes were expressed (RPKM > 0) in Δfst1 and WT, respectively, and significant differences were found in the expression of 367 genes. Of these, 147 (40.0%) genes were up-regulated in strain Δfst1 and 220 (60.0%) genes were down-regulated. In addition, we identified 39 and 19 genes that were uniquely expressed in WT and Δfst1, respectively. A previous study indicated that *FST1* is preferentially expressed in endosperm tissue relative to expression in germ [15]; therefore, we examined genes that encode secreted enzymes for starch and cell wall degradation, many that were previously described by Ravalason et al. [22]. Thirty-four differentially expressed genes were separated into five enzyme groups: cellulose-degrading, xylan-degrading, pectin-degrading, xylan/pectin-degrading and starch-degrading enzymes (Table 9). All groups contained genes that were affected (up- and down-regulated) by the mutation in strain Δfst1. Two genes with putative functions in starch degradation were expressed at reduced levels in strain Δfst1. We measured the expression of one of these, *AGD1* (FVEG_14136) by qPCR and verified its reduction (Table 6).

**Table 9:** **Differences in expression of putative, secreted, cell wall-degradation genes during colonization of autoclaved maize kernels by wild type (WT) and strain Δfst1** ^**a**^

**FVEG ID** ^**b**^	**Molecular function**	**P value** ^**c**^	**Log**_**2**_ **fold change** ^**d**^
	*Cellulose-degrading*		
05521	Glucosidase	0.0021	1.30
08733	Glycosidase	0.0010	1.35
09772	Glucosidase	0.0027	2.22
12965	Glycosidase	0.0004	1.93
13391	Glucosidase	0.0002	2.53
07232	Glucanase	0.0008	−1.32
01870	Glucosidase	0.0024	−1.80
11944	Glucanase	0.0009	−3.47
12142	Glucanase	0.0023	−1.74
13055	Glucosidase	0.0005	−1.31
12840	Glucanase	-	Δfst1^e^
10897	Glucanase	-	Δfst1
12594	Glucosidase	-	WT
	*Xylan-degrading*		
08344	Xylanase	0.0042	1.09
10098	Xylanase	0.0006	3.58
10625	Xylosidase	0.0006	1.69
12502	Xylanase	0.0006	1.19
13062	Xylanase	0.0055	1.18
13426	Xylosidase	0.0006	1.88
13578	Xylanase	0.0031	1.64
07261	Xylanase	0.0009	−1.42
13553	Xylanase	0.0068	−1.08
	*Pectin-degrading*		
04421	Galactosidase	0.0005	2.47
12299	Galactanase	8.3 E-06	2.02
08734	Pectic Lyase	0.0017	−2.76
11228	Pectinesterase	0.0095	−1.10
13516	Polygalacturonase	0.0023	−2.01
	*Xylan/pectin-degrading*		
05689	Arabinofuranosidase	0.0007	1.41
13426	Arabinofuranosidase	0.0006	1.88
07490	Glycosidase	2.2 E-06	−2.50
08421	arabinase	0.0004	−1.16
16349	Glycosidase	0.0002	−1.26
	*Starch-degrading*		
12681	Dextranase	0.0001	−1.62
14136	Glucosidase	9.4 E-05	−3.94

^a^Data were collected from cultures grown for 6 days.

^b^Fusarium Comparative Database (Broadinstitute.org).

^c^P value from pairwise t-test of mean RPKM of Δfst1 and WT.

^d^Values derived from the mean RPKM of Δfst1/WT.

^e^Transcript only detected in WT or Δfst1 as designated.

## Discussion

Previous studies indicated that deletion of *FST1* in *F. verticillioides* results in reduced fumonisin production and virulence [15,16]. Here we have linked the mutation to increased sensitivity to H_2_O_2_, reduced macroconidia production and reduced hydrophobicity. Considering these diverse phenotypes, the goal of this research was to characterize the effects of *FST1* on genome-wide expression during colonization of maize kernels. Autoclaved kernels were chosen to eliminate the effects associated with reduced biomass and fungal development caused by the slower growth of the *FST1* mutant when inoculated to living kernels. Even without a living host environment, significant changes in transcription were found in the mutant, many of which may contribute to the observed phenotypes.

For our comparison of the transcriptomes of WT and Δfst1, we relied on the *F. verticillioides* reference genome at the Broad Institute. Recent updates in the annotation of the genome created changes in gene reference numbers and gene identifications. Two changes were important to our study. First, the *FUM8* gene (originally: FVEG_00318, GenBank Accession No AAG27130) was separated into two genes: FVEG_14634 and 14635. In the original annotations, *FUM8* contained a 2532-bp open reading frame encoding a 839 amino acid protein described as the aminotransferase responsible for the condensation of alanine to the polyketide backbone of B-series fumonisins [23]. The disruption of *FUM8* in *F. verticillioides*, which blocks fumonisin production and mycotoxin production, was recovered in the mutant by complementation with the WT *FUM8* gene [23]. In the latest annotation of the genome, the sequence encoding the first 279 amino acids of FVEG_00318 plus 11 additional amino acids was designated as FVEG_14635, and the sequence encoding the last 554 amino acids of FVEG_00318, which contains aminotransferase domain, was designated as FVEG_14634. Regardless of this particular annotation error, expression of *FUM8* is significantly reduced in strain Δfst1 along with most of the other *FUM* genes, confirming the role of *FST1* in fumonisin production.

The second peculiar annotation change in the reference genome was that for *FST1* (FVEG_08441). Originally listed as a “hypothetical protein”, with similarity to hexose transporters, the gene is now listed as a “myo-inositol transporter”. Inositol is a polyol that functions as an essential constituent of cell membranes as derivatives of phosphatidylinositol and as important cell signaling molecules of inositol phosphates [24]. Two myo-inositol transporter genes have been described in *S. cerevisiae* by complementation of a strain defective in myo-inositol uptake [25]. A BLAST analysis of the *F. verticillioides* genome with the yeast ITR1p sequence identified eight genes with high sequence similarity (FVEG_01519, FVEG_01638, FVEG_02081, FVEG_03992, FVEG_06504, FVEG_07757, FVEG_11293, and FVEG_12687). The sequence of *FST1* was not identified by the search. Among the eight identified genes, expression was significantly down-regulated in Δfst1 for FVEG_06504 (named *ITR1*) (19-fold) and FVEG_03992 (5-fold), while the expression of FVEG_12687 was significantly up-regulated (12-fold). We measured the expression of *ITR1* by qPCR and verified that its expression was significantly reduced (Table 6). In light of these observations, the assignment of the functional role of myo-inositol transporter to *FST1* is premature.

Kim and Woloshuk [16] described the phenotype of Δfst1 as having slower growth and symptom development, and thus reduced virulence, compared to WT on wound-inoculated maize kernels. This growth inhibition was not observed on autoclaved kernels [15]. We hypothesized that the reduced virulence of Δfst1 resulted from an increased sensitivity to the effects of reactive oxygen species (ROS), which includes H_2_O_2_ produced by the living kernel [26,27]. The greater inhibition of the growth of strain Δfst1 by H_2_O_2_ compared to the WT and fst1-comp strains supports this hypothesis.

During pathogenesis, *F. verticillioides* could encounter ROS produced in maize kernels through several independent pathways. Kim and Woloshuk [16] inoculated the crown of maize kernels at the R4 (dough) stage of development, a period when the endosperm tissue is undergoing program cell death (PCD) [28]. ROS molecules, including H_2_O_2_, are produced during PCD in plants [29] and likely during endosperm development [30]. ROS production is also a characterized response of plants to pathogen invasion and plays a major role in host defense [31]. Most pathogens respond to ROS by the production of peroxidases and catalases [31]. Our RNAseq analysis of *F. verticillioides* grown on autoclaved kernels identified several putative catalases and peroxidases whose expression was changed in Δfst1 mutants. Four putative peroxidase genes were down-regulated in Δfst1, as were four of the six putative catalases. We also found that these oxidases were similarly affected in living kernels infected with the *F. verticillioides* strains.

To gain greater insight into a possible function of the catalases and how they may affect virulence, we examined their function in other plant pathogens. Catalases have been separated by phylogenetic analysis into four clades: peroxisomal, cytoplasmic, spore-specific, and secreted [32]. We found sequence similarity in the five differentially expressed catalases from our study when compared to the catalases assigned to the four clades in Giles [32]. FVEG_11955 was most similar to XP324526 in *Neurospora crassa* and FG02881 in *Gibberella zeae*, both of which belong to the peroxisomal catalase (clade P). FVEG_05976 was similar to FG05695 in *G. zeae*, which belongs to the cytoplasmic catalase (clade C). FVEG_05591 was similar to AAK15808 in *N. crassa* and FG06554 in *G. zeae*, which belong to the spore-specific catalase (clade A). Sequence analysis of the N-termini of the five predicted catalase proteins indicated that none are secreted.

As mentioned, catalases also have an important role in fungal development, including conidiogenesis. The sequences of the five differentially expressed, putative catalases in *F. verticillioides* are highly similar to *CATB* in *Magnaporthe grisea*, *CATA* and *CATB* in *A. nidulans*, *CAT1* and *CAT3* in *N. crassa*, and *CATB* in *Blumeria graminis*. In *M. grisea, CATB* is up regulated during infection of rice [33]. A strain disrupted in *CATB* was reduced in virulence with increased sensitivity to hydrogen peroxide, and was severely affected in conidia production. In addition, *CATA* mutants in *A. nidulans* exhibited reduction in conidiation and increased sensitivity to hydrogen peroxide [34]. The vast majority of conidial produced by *F. verticillioides* are microconidia. Although the number of macroconidia produced by the WT used in our study comprised only about 7% of the total conidia population, the reduction of macroconidia was consistently observed in strain Δfst1. From our study, it is not possible to determine if the altered expression of the five catalases in strain Δfst1 is responsible for the reduced production of macroconidia.

Aside from the role of catalases in conidial development, transcription factors are known to impact conidiation in fungi, and the expression of several putative TF genes were down-regulated in strain Δfst1. These genes include FVEG_16516 similar to *REN1* of *Fusarium oxysporum,* FVEG_09661 and FVEG_00646 similar to *BRLA* and *ABAA* of *A. nidulans*, respectively, FVEG_12826 similar to *FL* (*fluffy*) in *N. crassa*, and FVEG_06118 similar to FGSG_06160 in *F. graminearum.* Mutants of *REN1* and *ABAA* fail to produce normal conidia because of developmental malfunctions associated with phialides, the conidiogenous cells [35,36]. Mutants of *BRLA* fail to produce conidiophores [37] and *FL* mutants fail to produce conidia in chains [38]. Furthermore, expression of the conidiation-specific gene *CON-10* is not induced in *FL* mutants of *N. crassa*. In strain Δfst1, a gene (FVEG_00227) with high sequence identity to *CON-10* was down-regulated 14-fold compared to the WT. In *F. graminearum*, Son et al. [39] reported that deletion of FGSG_06160 results in a reduction in conidia production but no effect on virulence. We measured the expression of *FL*-like gene (*FLF1* FVEG_12826) by qPCR (Table 6). The expression was 2.9-fold of WT, which is near the 2.2-fold reduction obtained from the RNAseq analysis. These results indicate that reduced expression of one or more of these TFs may impact production of macroconidia but not microconidia.

Hydrophobins are another family of proteins that are associated with conidiogenesis as well as aerial hypha formation and have been shown to be involved in virulence [17,40-42]. Hydrophobins are separated into two classes based on spacing of cysteine residues and physical characteristics. Class I hydrophobins are highly insoluble proteins that form rodlets, and class II are more soluble and do not form rodlets. Fuchs et al. [17] predicted that hydrophobin genes *in F. verticillioides* encode three class I proteins (*HYD1* FVEG_03689, *HYD2* FVEG_03685 and *HYD3* FVEG_06538) and two class II proteins (*HYD4* FVEG_01575 and *HYD5* FVEG_07695). Examination of the protein sequences derived from *HYD6* (FVEG_01573) and *HYD8* (FVEG_10008) suggests they are class II and class I hydrophobins, respectively. We could not discern the class of *HYD7* (FVEG_09843) based on sequence alignments. Mutants of *F. verticillioides* with deletions of *HYD1* or *HYD2* are not defective in radial growth, conidial numbers, or corn seedling infection. However, these mutants fail to form microconidial chains [17]. Expression of these two genes was unaffected in Δfst1 and the strain produced normal microconidial chains. We observed the spreading of droplets of detergent solution placed on the surface of strain Δfst1, suggesting a deficiency in the more soluble class II hydrophobins [42]. The down-regulated expression of *HYD4*, *HYD5* and *HYD6* in strain Δfst1 is likely associated with this phenotype.

Previous studies have shown that fumonisin production and *FST1* expression are higher in the endosperm than in germ tissues [15,16]. These observations suggest that components within the endosperm provide an environment conducive for the pathogen. Strain Δfst1 grows as well as the WT on autoclaved maize, implying that it produces the secreted enzymes needed to breakdown macromolecules in the kernel and transporters to move nutrients into growing hyphae. However, our transcriptome results indicate that the mutation in *FST1* greatly impacts the expression of several genes that encode secreted enzymes. We found that the expression of genes encoding enzymes that degrade complex carbohydrate polymers, which make up host cell walls, was altered in strain Δfst1, but not uniformly. The lack of a growth phenotype in the mutant when grown on autoclaved maize and culture media may reflect functional redundancy in these large gene families [43]. For example, the expression of the alpha-amylase gene FVEG_12957 [15] was not affected in strain Δfst1. Expression of this gene would likely mask the potential effects caused by the down regulation of the two starch degradation genes (FVEG_12681 and FVEG_14136).

## Conclusion

In this study, we described three new phenotypes associated with a mutation in *FST1* that may contribute to the reduced virulence phenotype, namely the increased sensitivity to hydrogen peroxide, reduction of macroconidia production, and changes in mycelial hydrophobicity associated with Δfst1 mutants. We propose that reduced resistance to H_2_O_2_ in Δfst1 may impede the strain’s ability to respond to ROS encountered during pathogenesis. Our analysis of the transcriptomes of WT and Δfst1 indicated that the mutation of *FST1* affects the expression of 17% of the genes in *F. verticillioides*. Among the genes affected were many that impact mycotoxin biosynthesis, virulence, resistance to H_2_O_2_, and conidiogenesis. Our study supports the hypothesis that *FST1* has a role other than sugar transport. Other researchers have described putative sugar transporters that appear to have broader functions. Mutants of *RCO-3* in *N. crassa* displayed altered responses to increasing glucose concentrations in culture media [44]. The authors suggested that RCO-3 functions as a sugar sensor and a regulator of conidia production. In *Magnaporthe oryzae*, mutations affecting *MOST1* result in reduced conidiation and production of the secondary metabolite melanin [45]. The authors were not able to complement the defects by expression of other sugar transporter genes. Further studies are needed to determine how these genes (including *FST1*) regulate the function of multiple cell processes.

## Methods

### Fungal strains and culture conditions

*Fusarium verticillioides* strain 7600 (wild type, WT) is deposited in the Fungal Genetics Stock Center, University of Kansas Medical School, Kansas City, KS, USA. The mutant strain ∆fst1 and corresponding complemented stain fst1-comp were previously described by Bluhm et al. [15]. Cultures were stored long-term in 50% glycerol at −80°C and maintained as working stock on PDA medium (B&D, Sparks, MD).

### Phenotype assessment

To assess conidiation, strains were inoculated onto Petri dishes containing 1.5% water agar with six to eight gamma-irradiated carnation leaves (average size 18 mm^2^) on the agar surface [46]. For each fungal strain, nine carnation leaves were sampled after 7 days of incubation. Individual carnation leaves were placed into 1.5 ml microcentrifuge tubes containing 0.3 ml of water and vortexed briefly. Conidial number was determined with a hemacytometer [47]. Macroconidia and microconidia were recorded as the number of conidia per carnation leaf.

Resistance to hydrogen peroxide was measured as described by Lessing [48] and Ridenour [18] with some modifications. Conidia (1 ml of 1 × 10^6^ conidia) were mixed with 20 ml of molten PDA and poured into a Petri plate. After incubation for 24 hours at room temperature, a well was cut into the center of the plate with a cork borer (1 cm). To each well, 200 μl of 15% H_2_O_2_ (v/v) was added. Plates were incubated for another 24 hours at room temperature in the dark. Inhibition of growth appeared as a clear zone around the well. The area of the inhibition zone was determined. Test on each fungal strain was replicated three times.

Mycelial hydrophobicity was tested by placing droplets (10 μl) of water or a detergent solution (0.2% SDS, 50 mM EDTA) on the colony surface of strains grown on PDA medium for six days in the dark at room temperature. After 30 minutes, we determined whether or not the droplets maintained their spherical shape on the surface of the mycelium [18,49].

### Transcriptome analysis

Next-generation sequencing methods were used to obtain transcriptome data from the WT and strain ∆fst1 grown on autoclaved maize kernels. Kernels of maize inbred B73 were submerged in deionized water and autoclaved for 15 min. Afterwards, the kernels were crushed slightly to disrupt the pericarp, and approximately 7 g of kernels (10–12 kernels) were placed in glass vials (20 ml) and autoclaved for 30 min. Four replicate vials of the WT and ∆fst1 were inoculated with 100 μl of 10^6^ conidia/ml. Vials were incubated at 28°C for 6 days, then flash frozen in liquid nitrogen and stored at −80°C.

Total RNA was isolated from the content of each vial as described by Bluhm et al. [15] and purified with the RNeasy Mini Kit (Qiagen, Valencia, CA, USA). Further purification was achieved by treatment with the DNA-Free RNA kit (Zymo Research, Irvine, CA, USA). The Purdue Genetic Core Facility conducted quality assessment, processing, and sequencing of the RNA. The RNA samples had a RIN (RNA Integrity Number) over 7.0 as determined with an Agilent 2100 Bioanalyzer (Agilent Technologies, Palo Alto, CA, USA). Paired-end sequences were obtained with an Illumina HiSeq 2500 sequencer (Illumina, San Diego, CA, USA). Sequence data were trimmed of adapters and filtered to remove low quality sequence and reads less than 30 nt.

RNA sequence data from each sample were mapped to the reference genome of *Gibberella moniliformis*, which was downloaded (June 2014) from the Broad Institute Fusarium Comparative Database (http://www.broadinstitute.org). Sequence data were mapped to the reference genome was done with CLC Genomics Workbench (version 7.0.4, CLC Bio, Boston), and gene expression was quantified as reads per kilobase per million mapped reads (RPKM) [50]. Statistical analysis (pairwise t-testing) was also conducted with the CLC Genomics software. Differentially expressed genes between WT and ∆fst1 were sorted to identify those with absolute fold change values of > 2.0 and P value < 0.01. Genes expressed uniquely in each fungal strain were identified also. The selected genes from the differentially expressed and those in the uniquely expressed groups were analyzed for gene ontology (GO). For each gene, the translated sequence was analyzed with Blast2GO (version 2.7.2, Blast2Go.com). Results were sorted with respect to molecular function of the top BLAST descriptors.

### Quantitative real time-PCR

Quantitative PCR (qPCR) analysis was conducted on RNA isolated from both autoclaved and living maize kernels. For autoclaved kernels, equal amounts of purified RNA were pooled from the four biological replicates of WT and strain ∆fst1 used in the RNAseq anlaysis. To obtain living kernels, maize B73 was greenhouse-grown and ears were inoculated with the *F. verticillioides* strains as described by Kim and Woloshuk [16]. Six days after inoculation, infected kernels were collected from three ears (biological replicates) and total RNA was isolated. As with the autoclaved kernels, purified RNA were pooled from the three biological replicates of WT and strain ∆fst1.

cDNA was synthesized as described by Reese et al. [51]. Gene-specific PCR primers were designed with PrimerQuest Design Tool (Integrated DNA Technologies, Inc.) (Table 10). Quantitative PCR (qPCR) was conducted a described by Bluhm et al. [15] and reactions were replicated three times for each gene. Each reaction contained 1.5 μl of each primer pair (10 μM), 10 μl of iTaq Universal SYBR Green Supermix (Bio-rad, Hercules, CA), 5 μl of cDNA template, 2 μl of nuclease-free water. Reaction conditions were one cycle of 3 min at 95°C, 40 cycles of 5 s at 95°C and 30 s at 57°C. Expression of *TUB1* gene (FVEG_04081) was used to assure efficiencies of the target and reference reactions were approximately equal. The ∆∆Ct method [52] was used to calculate expression level with *TUB1* as the internal normalizer.

**Table 10:** **PCR primers used in this study**

**Primer Name**	**FVEG ID** ^**a**^	**Primer Sequence (5’ -> 3’)**
FST1-F	08441	CTT CTG ATG CTC TTC TCT TCC TCG C
FST1-R		TCT GGT ATA TCT CAC CAA TGA ACG CGA T
FUM1-F	00316	ACA CCA AAG CCT CTA CAA GTG A
FUM1-R		AGG TAT CGG GCA CCG CT
FUM21-F	14633	TTG CGA GGA TCT GTT CTT CTA TC
FUM21-R		TAT TAC CGA GCT TGC GCT ATA C
POD1-F	10866	TCA TTG ACC GTG CTC AAC TCC TCA
POD1-R		TGT CGA GTT GAC GAA GAA GT
POD3-F	12884	TCC TGG AAC AAC TGG AAT GG
POD3-R		CAA TCA AGA CAG ACA GGA GAG G
POD4-F	12465	GGC TAG CTA CAT CCA AGA AGA C
POD4-R		GTA CCA TCA GCC ATG ATC TCA A
CAT1-F	05529	GAT CTT CTG GAC CAA CCT CAA T
CAT1-R		CCT GAA CTT GGG CTC CTT ATA C
CAT2-F	12611	AGA AGA AGG CTG GTG CTA ATG
CAT2-R		GGC TCC ATG ACC TGA ACA TAC
CAT3-F	11955	GAG CGA CAC GCA AAC CAT TGA AGT
CAT3-R		ACC ACC AAC AGT CGA GAT TCG TGT
TUB1-F	04081	TGC TCA TTT CCA AGA TCC GCG
TUB1-R		GCG CAT GCA GAT ATC GTA GAG G
HYD3-F	06538	TTG CTC CAC CAA CTC TTA CTG
HYD3-R		GCG TTG ATG TTG ATG AGA GCA
HYD7-F	09843	AGC TCT CCG CCA TCT TCT A
HYD7-R		GCT CAA TGT CTC TCT CCT CAA C
ITR1-F	06504	GTC TCT CCC GTT CAT GAT TCT C
ITR1-R		GGG TTG ACT TGG GTG GTA TT
FLF1-F	12826	AGC GAT GCT TCT TGT CCT TAC
FLF1-R		AAC CAA GCT CAC GAC CTA TTT
TFS1-F	06118	GGG ACC TGT TGC CAT TAA GA
TFS1-R		TCA TCC TCC GGC ATT TCA TAG
AGD1-F	14136	CGT ATG GCA GAG TGG GTA AAT
AGD1-R		CAT CAG GAT TCG GAC GGT ATA TG

^a^Fusarium Comparative Database (Broadinstitute.org).

### Availability of supporting data

Supporting sequence data are available in NCBI’s Gene Expression Omnibus and are accessible through GEO Series accession number GSE66044 (http://www.ncbi.nlm.nih.gov/geo/query/acc.cgi?acc=GSE66044).

## References

[1] Oren L, Ezrati S, Cohen D, Sharon A (2003). Early events in the *Fusarium verticillioides*–maize interaction characterized by using a green fluorescent protein–expressing transgenic isolate. Appl Environ Microbiol.

[2] Hendricks K (1999). Fumonisins and neural tube defects in South Texas. Epidemiology.

[3] Wu F (2006). Mycotoxin reduction in Bt corn: potential economic, health, and regulatory impacts. Transgenic Res.

[4] Wu F (2004). Mycotoxin risk assessment for the purpose of setting international regulatory standards. Environ Sci Technol.

[5] Brown DW, Butchko RA, Busman M, Proctor RH (2007). The *Fusarium verticillioides FUM* gene cluster encodes a Zn(II)2Cys6 protein that affects *FUM* gene expression and fumonisin production. Eukaryot Cell.

[6] Visentin I, Montis V, Doll K, Alabouvette C, Tamietti G, Karlovsky P (2012). Transcription of genes in the biosynthetic pathway for fumonisin mycotoxins is epigenetically and differentially regulated in the fungal maize pathogen *Fusarium verticillioides*. Eukaryot Cell.

[7] Liu SY, Lin JQ, Wu HL, Wang CC, Huang SJ, Luo YF, *et al.* Bisulfite sequencing reveals that *Aspergillus flavus* holds a hollow in DNA methylation. *PloS One* 2012, 7(1). 10.1371/journal.pone.0030349 PMC3262820 22276181

[8] Woloshuk CP, Shim WB (2013). Aflatoxins, fumonisins, and trichothecenes: a convergence of knowledge. FEMS Microbiol Rev.

[9] Kim H, Woloshuk CP (2008). Role of *AREA*, a regulator of nitrogen metabolism, during colonization of maize kernels and fumonisin biosynthesis in *Fusarium verticillioides*. Fungal Genet Biol.

[10] Shim WB, Woloshuk CP (2001). Regulation of fumonisin B–1 biosynthesis and conidiation in *Fusarium verticillioides* by a cyclin–like (C–type) gene, FCC1. Appl and Environ Microbiol.

[11] Tilburn J, Arst HN, Penalva MA. Regulation of gene expression by ambient pH. Cell Mol Biol Filamentous Fungi 2010:480--487. 10.1128/MMBR.66.3.426-446.2002 PMC120796 12208998

[12] Flaherty JE, Pirttila AM, Bluhm BH, Woloshuk CP (2003). PAC1, a pH–regulatory gene from *Fusarium verticillioides*. Appl and Environ Microbiol.

[13] Bluhm BH, Woloshuk CP (2005). Amylopectin induces fumonisin B–1 production by *Fusarium verticillioides* during colonization of maize kernels. Mol Plant Microbe In.

[14] Kim H, Smith JE, Ridenour JB, Woloshuk CP, Bluhm BH (2011). *HXK1* regulates carbon catabolism, sporulation, fumonisin B(1) production and pathogenesis in *Fusarium verticillioides*. Microbiol--Sgm.

[15] Bluhm BH, Kim H, Butchko RAE, Woloshuk CP (2008). Involvement of *ZFR1* of *Fusarium verticillioides* in kernel colonization and the regulation of *FST1*, a putative sugar transporter gene required for fumonisin biosynthesis on maize kernels. Mol Plant Pathol.

[16] Kim H, Woloshuk CP (2011). Functional characterization of *fst1* in *Fusarium verticillioides* during colonization of maize kernels. Mol Plant Microbe In.

[17] Fuchs U, Czymmek KJ, Sweigard JA (2004). Five hydrophobin genes in *Fusarium verticillioides* include two required for microconidial chain formation. Fungal Genet Biol.

[18] Ridenour JB, Bluhm BH (2014). The HAP complex in *Fusarium verticillioides* is a key regulator of growth, morphogenesis, secondary metabolism, and pathogenesis. Fungal Genet Biol.

[19] Ma LJ, van der Does HC, Borkovich KA, Coleman JJ, Daboussi MJ, Di Pietro A (2010). Comparative genomics reveals mobile pathogenicity chromosomes in *Fusarium*. Nature.

[20] Wiemann P, Sieber CMK, Von Bargen KW, Studt L, Niehaus EM, Espino JJ, *et al.*: Deciphering the cryptic genome: genome--wide analyses of the rice pathogen *Fusarium fujikuroi* reveal complex regulation of secondary metabolism and novel metabolites. *Plos Pathog* 2013, 9(6). 10.1371/journal.ppat.1003475 PMC3694855 23825955

[21] Park J, Park J, Jang S, Kim S, Kong S, Choi J (2008). FTFD: an informatics pipeline supporting phylogenomic analysis of fungal transcription factors. Bioinformatics.

[22] Ravalason H, Grisel S, Chevret D, Favel A, Berrin JG, Sigoillot JC (2012). Fusarium verticillioides secretome as a source of auxiliary enzymes to enhance saccharification of wheat straw. Bioresource Technol.

[23] Seo JA, Proctor RH, Plattner RD (2001). Characterization of four clustered and coregulated genes associated with fumonisin biosynthesis in *Fusarium verticillioides*. Fungal Genet Biol.

[24] Barker CJ, Illies C, Gaboardi GC, Berggren PO (2009). Inositol pyrophosphates: structure, enzymology and function. Cell Mol Life Sci.

[25] Nikawa J, Tsukagoshi Y, Yamashita S (1991). Isolation and characterization of two distinct myo–inositol transporter genes of *Saccharomyces cerevisiae*. J Biol Chem.

[26] Torres MA, Jones JD, Dangl JL (2006). Reactive oxygen species signaling in response to pathogens. Plant Physiol.

[27] Heller J, Tudzynski P (2011). Reactive oxygen species in phytopathogenic fungi: signaling, development, and disease. Annu Rev Phytopathol.

[28] Young TE, Gallie DR (2000). Programmed cell death during endosperm development. Plant Mol Biol.

[29] Van Breusegem F, Dat JF (2006). Reactive oxygen species in plant cell death. Plant Physiol.

[30] Sabelli PA (2012). Replicate and die for your own good: endoreduplication and cell death in the cereal endosperm. J Cereal Sci.

[31] Torres MA (2010). ROS in biotic interactions. Physiol Plant.

[32] Giles SS, Stajich JE, Nichols C, Gerrald QD, Alspaugh JA, Dietrich F (2006). The *Cryptococcus neoformans* catalase gene family and its role in antioxidant defense. Eukaryot Cell.

[33] Skamnioti P, Henderson C, Zhang ZG, Robinson Z, Gurr SJ (2007). A novel role for catalase B in the maintenance of fungal cell–wall integrity during host invasion in the rice blast fungus *Magnaporthe grisea*. Mol Plant Microbe In.

[34] Navarro RE, Stringer MA, Hansberg W, Timberlake WE, Aguirre J (1996). *catA*, a new *Aspergillus nidulans* gene encoding a developmentally regulated catalase. Cur Genet.

[35] Ohara T, Inoue I, Namiki F, Kunoh H, Tsuge T (2004). REN1 is required for development of microconidia and macroconidia, but not of chlamydospores, in the plant pathogenic fungus *Fusarium oxysporum*. Genetics.

[36] Sewall TC, Mims CW, Timberlake WE (1990). Abaa controls phialide differentiation in *Aspergillus–nidulans*. Plant Cell.

[37] Adams TH, Boylan MT, Timberlake WE (1988). brlA is necessary and sufficient to direct conidiophore development in *Aspergillus nidulans*. Cell.

[38] Bailey LA, Ebbole DJ (1998). The fluffy gene of *Neurospora crassa* encodes a Gal4p–type C6 zinc cluster protein required for conidial development. Genetics.

[39] Son H, Seo YS, Min K, Park AR, Lee J, Jin JM, et al. A phenome--based functional analysis of transcription factors in the cereal head blight fungus, *Fusarium graminearum*. Plos Pathog 2011;7(10):e1002310 10.1371/journal.ppat.1002310 PMC3197617 22028654

[40] Talbot NJ, Kershaw MJ, Wakley GE, de Vries OMH, Wessels JGH, Hamer JE (1996). MPG1 encodes a fungal hydrophobin involved in surface interactions during infection–related development of *Magnaporthe grisea*. Plant Cell.

[41] Wosten HAB (2001). Hydrophobins: multipurpose proteins. Annu Rev Microbiol.

[42] Wosten HAB, Devries OMH, Wessels JGH (1993). Interfacial self–assembly of a fungal hydrophobin into a hydrophobic rodlet layer. Plant Cell.

[43] Wagner A (2005). Distributed robustness versus redundancy as causes of mutational robustness. Bioessays.

[44] Madi L, McBride SA, Bailey LA, Ebbole DJ (1997). rco–3, a gene involved in glucose transport and conidiation in *Neurospora crassa*. Genetics.

[45] Saitoh H, Hirabuchi A, Fujisawa S, Mitsuoka C, Terauchi R, Takano Y (2014). MoST1 encoding a hexose transporter–like protein is involved in both conidiation and mycelial melanization of *Magnaporthe oryzae*. FEMS Microbiol Let.

[46] Fisher NL, Burgess LW, Toussoun TA, Nelson PE (1982). Carnation leaves as a substrate and for preserving cultures of *Fusarium* species. Phytopathology.

[47] Aberkane A, Cuenca–Estrella M, Gomez–Lopez A, Petrikkou E, Mellado E, Monzon A (2002). Comparative evaluation of two different methods of inoculum preparation for antifungal susceptibility testing of filamentous fungi. J Antimicrob Chemother.

[48] Lessing F, Kniemeyer O, Wozniok I, Loeffler J, Kurzai O, Haertl A (2007). The *Aspergillus fumigatus* transcriptional regulator *AfYap1* represents the major regulator for defense against reactive oxygen intermediates but is dispensable for pathogenicity in an intranasal mouse infection model. Eukaryot Cell.

[49] Yan X, Li Y, Yue XF, Wang CC, Que YW, Kong DD, et al. Two novel transcriptional regulators are essential for infection--related morphogenesis and pathogenicity of the rice blast fungus *Magnaporthe oryzae*. Plos Pathog 2011;7(12):e1002385 10.1371/journal.ppat.1002385 PMC3228794 22144889

[50] Mortazavi A, Williams BA, McCue K, Schaeffer L, Wold B (2008). Mapping and quantifying mammalian transcriptomes by RNA–Seq. Nat Methods.

[51] Reese BN, Payne GA, Nielsen DM, Woloshuk CP (2011). Gene expression profile and response to maize kernels by *Aspergillus flavus*. Phytopathology.

[52] Livak KJ, Schmittgen TD (2001). Analysis of relative gene expression data using real–time quantitative PCR and the 2(Delta Delta C(T)) Method. Methods.

